# A database of flavivirus RNA structures with a search algorithm for pseudoknots and triple base interactions

**DOI:** 10.1093/bioinformatics/btaa759

**Published:** 2020-08-31

**Authors:** Alan Zammit, Leon Helwerda, René C L Olsthoorn, Fons J Verbeek, Alexander P Gultyaev

**Affiliations:** Group Imaging & Bioinformatics, Leiden Institute of Advanced Computer Science (LIACS), Leiden University, 2300 RA Leiden, The Netherlands; Group Imaging & Bioinformatics, Leiden Institute of Advanced Computer Science (LIACS), Leiden University, 2300 RA Leiden, The Netherlands; Group Supramolecular & Biomaterials Chemistry, Leiden Institute of Chemistry, Leiden University, 2300 RA Leiden, The Netherlands; Group Imaging & Bioinformatics, Leiden Institute of Advanced Computer Science (LIACS), Leiden University, 2300 RA Leiden, The Netherlands; Group Imaging & Bioinformatics, Leiden Institute of Advanced Computer Science (LIACS), Leiden University, 2300 RA Leiden, The Netherlands; Department of Viroscience, Erasmus Medical Center, Rotterdam, 3000 CA, The Netherlands

## Abstract

**Motivation:**

The *Flavivirus* genus includes several important pathogens, such as Zika, dengue and yellow fever virus. Flavivirus RNA genomes contain a number of functionally important structures in their 3′ untranslated regions (3′UTRs). Due to the diversity of sequences and topologies of these structures, their identification is often difficult. In contrast, predictions of such structures are important for understanding of flavivirus replication cycles and development of antiviral strategies.

**Results:**

We have developed an algorithm for structured pattern search in RNA sequences, including secondary structures, pseudoknots and triple base interactions. Using the data on known conserved flavivirus 3′UTR structures, we constructed structural descriptors which covered the diversity of patterns in these motifs. The descriptors and the search algorithm were used for the construction of a database of flavivirus 3′UTR structures. Validating this approach, we identified a number of domains matching a general pattern of exoribonuclease Xrn1-resistant RNAs in the growing group of insect-specific flaviviruses.

**Availability and implementation:**

The Leiden Flavivirus RNA Structure Database is available at https://rna.liacs.nl. The search algorithm is available at https://github.com/LeidenRNA/SRHS.

**Supplementary information:**

Supplementary data are available at *Bioinformatics* online.

## 1. Introduction

The Flavivirus genus includes several species responsible for serious human diseases, such as Zika fever, dengue, Japanese encephalitis, yellow fever and others. Flaviviruses are mostly arthropod-borne (arboviruses), thus able to complete their life cycles in arthropods like mosquitoes and ticks. Infections of humans and other animals happen by arthropod blood-sucking behavior. Certain flaviviruses cause millions of infections per year worldwide and are considered to be emerging viruses (Mackenzie *et al*., 2004).

**Fig. 1.**
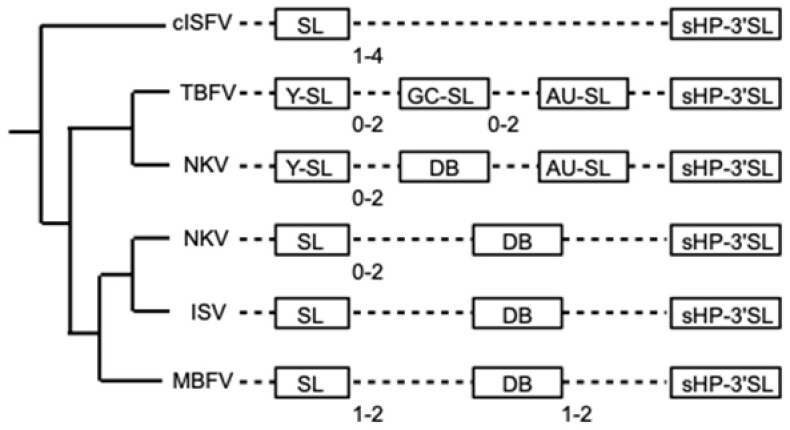
Locations of the main conserved structural elements in flavivirus 3′UTRs (reviewed by Villordo *et al.*, 2016): stem-loop (SL), Y-shaped stem-loop (Y-SL), dumbbell structure (DB), GC-containing stem-loop (GC-SL), AU-containing stem-loop (AU-SL) and small hairpin—3′stem-loop (sHP-3′SL). Duplications of structural elements are indicated with the ranges of repeat numbers. Phylogenetic tree of the major flavivirus clades is schematically shown

Higher-order structures of flavivirus positive-sense RNA genomes are essential for virus replication (reviewed by Fernandez-Sanles *et al*., 2017). In particular, relatively large (400–900 nucleotides) 3′ untranslated regions (3′UTRs) of flavivirus genomes contain a number of functionally important structured elements that can be classified in several topological classes (Gritsun and Gould, 2007; MacFadden *et al*., 2018; Olsthoorn and Bol, 2001; Villordo *et al*., 2016). Diversity of the motif topologies mostly follows phylogenetic classification of flaviviruses into the main ecological groups: mosquito-borne flaviviruses (MBFV), tick-borne flaviviruses (TBFV), vertebrate flaviviruses with no known vector (NKV) and insect-specific flaviviruses (ISFV), some of the latter cluster in a separate group of so-called classic insect-specific flaviviruses (cISFV)(Fig. 1). There are also some differences between structures in virus subgroups and diverse arrangements of these structural elements with frequent duplications seem to be related to virus adaptation for different hosts (de Borba *et al*., 2019; Villordo *et al.*, 2016). The presence of domains resistant to progression of the 5′→3′ degradation by exoribonuclease Xrn1 (xrRNAs) in the 5′-proximal parts of flavivirus 3′UTRs determines production of subgenomic flaviviral RNAs (sfRNAs), which contain the downstream 3′UTR parts and are important virulence factors (Göertz *et al*., 2016; Moon *et al*., 2015; Pijlman *et al*., 2008; Schnettler *et al*., 2012).

The conserved motifs include stem-loop secondary structures, pseudoknots and triple base interactions (Akiyama *et al*., 2016; Chapman *et al*., 2014; Gritsun and Gould, 2007; MacFadden *et al.*, 2018; Olsthoorn and Bol, 2001; Villordo *et al.*, 2016). Such complex topologies in combination with high sequence diversity make the search for sites satisfying these motifs in flavivirus genomes rather difficult. Straightforward sequence alignment comparisons are only helpful in cases of sufficient sequence similarity. Thermodynamics-based structure predictions mostly ignore pseudoknot formation, which leads to problems in unequivocally recognizing even the elements of the orthodox RNA secondary structure in flavivirus 3′UTRs (Andrews *et al*., 2018). With covariance models (CMs), an efficient tool for the description of structured RNA families (Kalvari *et al*., 2018; Nawrocki *et al*., 2009; Nawrocki and Eddy, 2013a), it is possible to identify some elements of orthodox secondary structure in flavivirus UTR (https://rfam.org; Ochsenreiter *et al*., 2019). However, covariance models do not include pseudoknots either and thus consider only parts of flavivirus RNA structures ignoring essential functional elements. In contrast, available algorithms for pseudoknot searching with training on sequence-structure alignments (Cai *et al*., 2003; Gautheret and Lambert, 2001; Huang *et al*., 2008; Lambert *et al*., 2004) would not be practical in case of flavivirus RNA motifs because of complexity of structures and sequence diversity. For instance, such programs need certain approximations like the presence of gaps only in the loops (Gautheret and Lambert, 2001) or they can miss structure matches due to variations in numbers of canonical base pairs in homologous stems, e.g. in tmRNAs (Huang *et al.*, 2008). Furthermore, these approaches require high-quality multiple sequence-structure alignments, which are difficult to obtain for 3′UTRs of distant flaviviruses in the absence of structural information. The alignment limitation is only partially applicable for a descriptor-based approach, because after the descriptor construction using local alignments of separate elements the searches could be executed across the whole 3′UTR lengths even in cases of considerable sequence divergence and motif duplications that create alignment problems.

A knowledge gap between the number of sequenced flavivirus genomes and annotation of their 3′UTR structures is steadily increasing due to continuous discovery of novel flaviviruses that infect diverse ranges of species and are distantly related to each other (e.g. Blitvich and Firth, 2015; Parry and Asgari, 2019; Skoge *et al*., 2018). Given the importance of flavivirus 3′UTR structures and dangers of emerging and re-emerging flavivirus pathogens (e.g. Gould *et al*., 2017; Mackenzie *et al.*, 2004), it is important to compile available data in a systematic way and develop techniques for efficient identification of functional structures in novel viruses and strains. These techniques should be implemented as tools suitable for researchers that are not advanced computer users and do not have extensive knowledge of RNA folding principles.

In general, the problem of searching for pseudoknot-containing structures can be solved by a threading of sequences with descriptors that define the structural patterns of motifs in question (Gautheret *et al*., 1990; Macke, 2001; Riccitelli and Lupták, 2010). Here, we introduce a database of flavivirus 3′UTR structural motifs, including pseudoknots and triple base contacts. The motifs were compiled in the form of structural descriptors, which were derived from the available data on functional interactions in flavivirus RNA 3′UTRs. An algorithm for searching for descriptor-matching sequences was developed. Testing the algorithm showed that it is an efficient tool for detecting similar structural patterns in diverse flavivirus genome sequences, in particular, those that are important for identification of potential subgenomic RNAs.

## 2 Materials and methods

### 2.1 RNA structure descriptor format

**Fig. 2.**
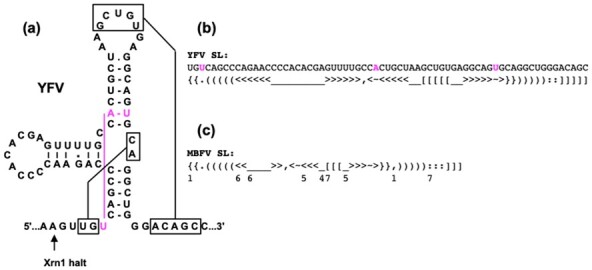
An example of WUSS representation of RNA structure containing pseudoknots and a triple base interaction. (**a**) The structure of Xrn1-resistant stem-loop RNA structure (xrRNA) of yellow fever virus (YFV) according to the general model (MacFadden *et al.*, 2018). Paired regions in pseudoknots are boxed, triple base interaction is shown in magenta. The point of Xrn1 halt (Silva et al., 2010) is indicated by arrow. (**b**) Structural annotation of the YFV SL domain using WUSS symbols. Dot and tildes indicate a triple base interaction. (**c**) The general descriptor for MBFV xrRNA SL domains. A number below a symbol in the descriptor indicates potential multiplication of the symbol in the searched structures. For instance, 5 below a symbol for base pair means that a this site 0, 1, 2, 3, 4 or 5 base pairs are allowed

RNA structure descriptors were based on ‘WUSS notation’ (Washington University Secondary Structure notation), also used in the Infernal and the Rfam database (Kalvari *et al.*, 2018; Nawrocki and Eddy, 2013b). This is a linear string representation of secondary structure elements with bracket pairs corresponding to paired nucleotide positions. WUSS notation extends the simpler bracket view of RNA secondary structure, used in a number of tools, with different types of symbols denoting the base pairs and unpaired nucleotides involved in different topological parts of RNA structure. The WUSS notation is easier for human perception than simplified bracket views and allows an unambiguous annotation of pseudoknots. For a triple base interaction, we introduced additional notation with a dot and two tildes, tildes being used for paired nucleotides within a helix. The symbols used are illustrated in Figure 2 with the MBFV SL structure as an example.

### 2.2 Consensus secondary structure descriptor design

Initially, flavivirus 3′UTR RNA structure descriptors were designed for the main classes of flavivirus 3′UTR structural elements (reviewed by Villordo *et al.*, 2016), namely, MBFV stem-loop (SL) and dumbbell (DB) structures and TBFV Y-SL, GC-SL and AU-SL structures. For definitions of structural constraints and the ranges of variations in the sizes of stems and loops, we used the models published for the 3′UTRs of MBFV, TBFV, NKV and ISFV (Gritsun and Gould, 2007; MacFadden *et al.*, 2018; Olsthoorn and Bol, 2001; Schnettler *et al*., 2014; Villordo *et al.*, 2016). The flavivirus RNA sequences used for the design of descriptors are shown in Supplementary Table S1. Although the secondary structure models for Xrn1-resistant Y-SL elements of TBFV and NKV (MacFadden *et al.*, 2018; Schnettler *et al.*, 2014; Villordo *et al.*, 2016) deviate from the Xrn1-resistant MBFV SL structures, our sequence alignments suggest the presence of homologous tertiary structure elements, such as double pseudoknot and the triple base interaction (manuscript in preparation); these were included in both the MBFV SL and TBFV/NKV Y-SL descriptors. Furthermore, alignment of homologous structures predicted by these descriptors in the 3′UTRs of the separate clade of classic insect-specific viruses (cISFV) (Blitvich and Firth, 2015; Colmant *et al*., 2017) allowed us to construct specific xrRNA descriptors for this group.

### 2.3 Search algorithm

**Fig. 3.**
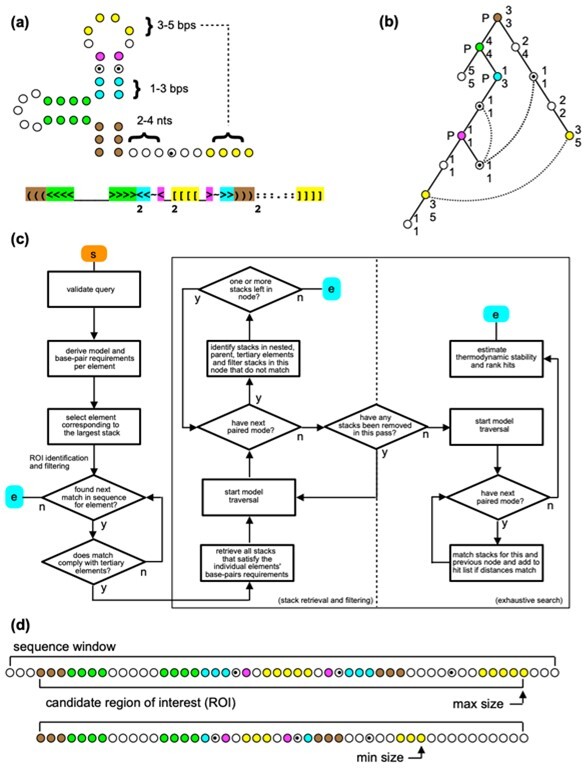
Model construction and sequence querying. (**a**) Exemplary structure with tertiary interactions and the corresponding CSSD in extended WUSS notation. (**b**) Tree-like model, derived from CSSD. The model built from unpaired (U) and paired (P) node types, the latter of which (flanked by P) relate to orthodox secondary structure. Tertiary interactions are captured using U-node associations (dashed lines). Min/max sizes are shown for both node types. (**c**) Schematic for sequence querying. Sequence regions of interest (ROIs) are identified, each seeded by locating strands compatible with the largest base-paired element (in this example, green). Iterating over all node sizes, distances from this element to pseudoknot (yellow) and triple base (dotted) elements are used to validate each ROI. Processed in parallel, each ROI is then scanned for base pair stacks that are assigned to the respective P-nodes. Each node's stack is pairwise-tested against immediately nested and parent structures as well as tertiary interactions. In a final step, any remaining stacks are validated exhaustively using a pre-order, depth-first model traversal with backtracking. During traversal, stacks from newly visited P-nodes are matched distance-wise against stacks from previous nodes. U-nodes associated with tertiary elements are only tested once the most 3′ node is reached. Any resulting hits are ranked based on thermodynamic stability estimates. (**d**) Min/max structures expressed by this model, in context of a candidate ROI

Threading sequences through complex and lenient RNA structural patterns may incur considerable runtime costs (e.g. Gautheret *et al.*, 1990; Riccitelli and Lupták, 2010). Due to considerable sequence diversity of flavivirus RNA structures, we did not try to solve this problem by any sequence restrictions in the designed descriptors, except for those imposed by base pairing and triple base interactions. In the approach adopted here, high runtime costs were curtailed using parallelism, stepwise filtering of potential structural elements and adopting elements of dynamic programming when executing searches for matching sequences (Fig. 3).

Here, a consensus secondary structure descriptor (CSSD) **C** representing a target RNA motif is defined by two character strings. The first one contains WUSS symbols for base pairing information and additional symbols specifying triple base interactions. In case of flavivirus xrRNA motifs, the triple base interactions constrained the matches by isosteric triples C.G-C (or C.G-U) and U.A-U (MacFadden *et al.*, 2018). The second string holds individual alphanumeric characters, denoting size (length) variabilities of all stems and loops in the first string in extended WUSS format (Figs 2 and 3a).

In the parsing of a descriptor, a binary tree structure **T** is constructed using two types of nodes: paired nodes (P-nodes) and unpaired nodes (U-nodes), with both types retaining information about variability in size. An example of tree construction is given in Figure 3b. P-nodes denote instances of uninterrupted regular helices in **C**, whereas U-nodes represent contiguous stretches of unpaired nucleotides. A P-node shares one edge, here referred to as its 5′ link, with another adjacent U- or P-node of the element in **C** that is nested within the P-node's base-paired structure. A second, optional edge between the same P-node and another node, its 3′ link, signals adjacency to a downstream structural element. A U-node may only have one edge with another U- or P-node. A vacant U-node link or a vacant P-node 3′ link, terminates the tree. By way of example, a hairpin structure can be represented by a two-node tree with a P-node (stem) 5′ linked to a terminating U-node (loop). Tertiary structure elements such as pseudoknots and triple base interactions are incorporated into the basic tree structure by cross-linking specially defined U-node types. Using this modeling approach, motifs involving complex structural elements can efficiently be visited in a single sweep of **T**.

The search in a query sequence **S** starts from filtering any potential regions of interest (Fig. 3d). The initial filtering step retrieves segments in **S** that satisfy a P-node in **T** with the largest size (P'). Note that after traversing **T**, the information on minimum and maximum sizes of all elements on both sides of P' and on their distances from P' are readily available. Next, **S** is scanned to identify the segments where all structural elements of **T** are matched at least once within the defined ranges of sizes and distances. Each candidate region (R) of **S** is subsequently processed in parallel with filtering of compatible base pairs. First, for every P-node in **T**, and all sizes of that node, all matching sets of base pairs (stacks) in **R** are enlisted. Each of these sets is then tested for the following requirements: (i) the stack is compatible with tertiary structural constraints defined in **C**, (ii) the stack matches at least one other stack that is structurally nested within (contained) and (iii) the stack matches at least one other stack that contains that set.

Using sets of base pairs available from the preceding filtering step, and assigning them to individual nodes, **T** is traversed recursively while iterating over all sizes prescribed by each P- and U-node. While traversing the model, both size and linking requirements of the nodes must be satisfied. As a search proceeds recursively, candidate base pairs associated with the current iteration of a P-node are validated against any base pairs made available by the preceding P-node. This procedure yields the structures matching **C** within **R**.

Larger CSSDs, when in the order of hundreds of millions and up to a few billion unique consensa, are likely to produce several multiple hits at the same site due to combinatorial diversity of similar structures with variations in sizes of their elements. For practical reasons, a configurable upper limit on the number of hits returned per region of interest was set, currently 500. This relatively high value ensures that search accuracy is not compromised, given that sorting by stacking free energy filters out less stable structures while more stable functional ones are yielded to a user.

### 2.4 Estimates of thermodynamic stability of predicted structures

Stacking free energies in double-helical stems, including pseudoknotted ones, were calculated according to Turner 2004 parameters, compiled in the Nearest Neighbor Database (NNDB) (Turner and Mathews, 2010). Coaxial stacking between stems P1 and P2 in the MBFV SL structures, revealed by crystallographic data (Akiyama *et al.*, 2016; Chapman *et al.*, 2014), was also considered with the values equal to those in regular helices (Turner and Mathews, 2010). The sum of free energies in all stacks of a given structure was used to estimate its thermodynamic stability.

### 2.5 Implementation

The algorithm was implemented in C as a loosely coupled, cross-platform, multi-threaded application, with support for MPI-enabled compute clusters. A number of third-party, open-source components were used and integrated into the system, such as Bootstrap, Knockout (web framework), jQuery, GNU libmicrohttpd, Ulfius (REST framework), Jansson, jemalloc (memory allocator), MongoDB, Open MPI, TORQUE, Slurm. Standard tools and frameworks were used to provide a web front-end to the database. The database of flavivirus 3′UTR structures was implemented as a free to use service and is available at the Leiden Flavivirus RNA Structure Database Server (https://rna.liacs.nl).

## 3. Results

**Fig. 4.**
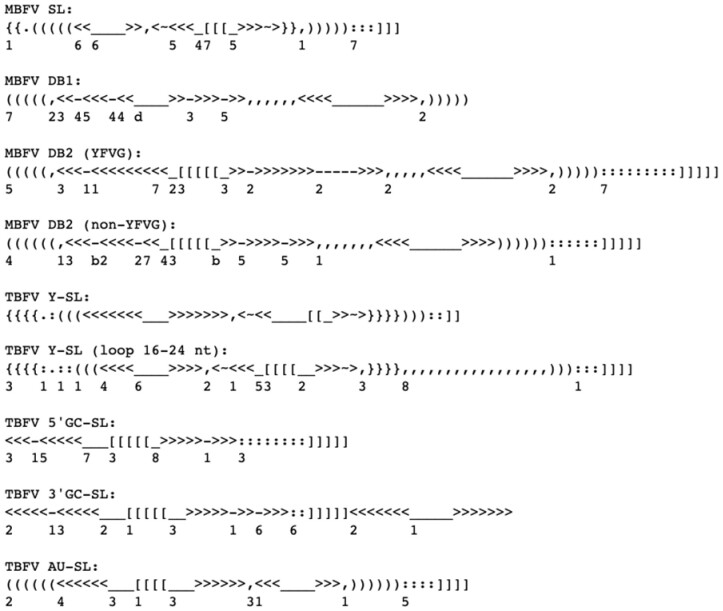
The designed structure descriptors of conserved structures in flaviviruses. In addition to digits, alphanumeric characters are used for size ranges (*a* = 10; *b* = 11; etc.). YFVG, yellow fever virus group. Other abbreviations are given in Fig. 1 legend

Published structural models of conserved structures in the 3′UTRs of MBFV, TBFV, NKV and ISFV allowed us to construct structural descriptors which could serve as consensus motifs for different types of structures (reviewed by Villordo *et al.*, 2016). Testing the behavior of the search algorithm showed that a general descriptor for all MBFV dumbbell (DB) structures would define large numbers of consensa, tens of billions or more, leading to an unfavorable computational and memory footprint. To avoid this, three DB descriptors were used: one for the upstream DB1 without account for the long-range pseudoknot, and two for the duplicated DB2 with a pseudoknot, one of which being specific for yellow fever virus group. Similarly, the descriptor for Xrn1-resistant TBFV Y-SL structures was split into two, with the main topological difference being in the size ranges of one of the loops. This splitting allowed us to define general descriptors for xrRNA structures in TBFV, NKV and ISFV 3′UTRs. In total, nine descriptors (Fig. 4) turned out to be sufficient for the search for the known structures with reasonable computation times.

**Fig. 5.**
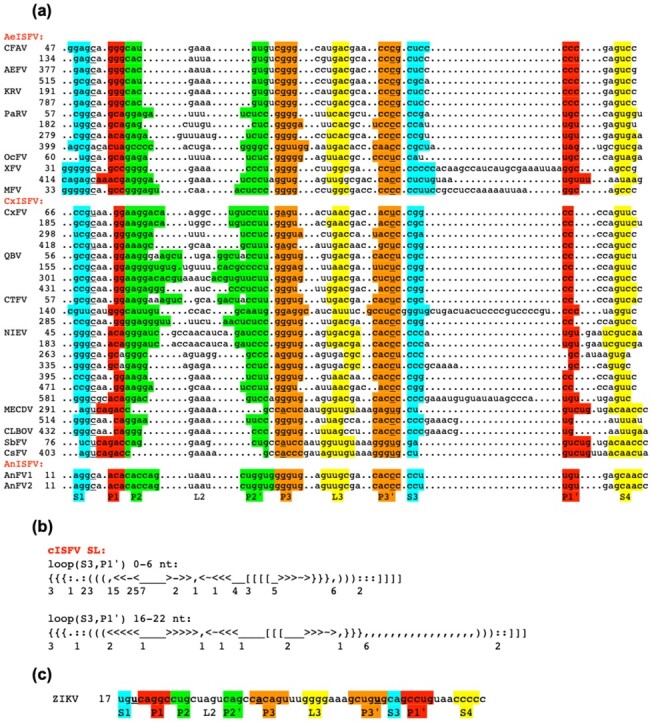
Predicted xrRNA structures in the 3′UTRs of flaviviruses of the cISFV group. (**a**) The paired regions are denoted with specific colours. For the sake of clarity, the pseudoknot interactions are not aligned. The notations for the secondary structure elements below the sequences are taken from the general model for Xrn1-resistant flavivirus SL structures (Akiyama *et al.*, 2016; MacFadden *et al.*, 2018). Bases involved in the conserved triple interactions are in bold and underlined. Numbering begins from the 5′ends of 3′UTRs. (**b**) The consensus descriptor on the basis of predicted cISFV xrRNA elements was constructed in two versions which differed mostly in size ranges of the loop between S3 and P1′ elements. (**c**) Homologous structure of the Zika virus (ZIKV) 3′UTR (Akiyama *et al.*, 2016) is shown for comparison. Abbreviations and accession numbers: AeISFV, CxISFV and AnISFV, *Aedes*-, *Culex* and *Anopheles*-associated insect-specific flaviviruses; CFAV, Cell fusing agent virus (accession NC_001564); AEFV, Aedes flavivirus (NC_012932); KRV, Kamiti River virus (NC_005064); PaRV, Paramatta River virus (KT192549); OcFV, Ochlerotatus caspius flavivirus (NC_034242); XFV, Xishuangbanna aedes flavivirus (NC_034017); MFV, Menghai flavivirus (NC_034204); CxFV, Culex flavivirus (NC_008604); QBV, Quang Binh virus (NC_012671); CTFV, Culex theileri virus (HE574574); NIEV, Nienokoue virus (NC_024299); MECDV. Mercadeo virus (KP688057); CLBOV, Calbertado virus (KX669688); SbFV, Sabethes flavivirus (MH899446); CsFV, Culiseta flavivirus (KT599442); AnFV1 and AnfV2, Anopheles flavivirus 1 and 2 (KX148546 and KX148547)

The descriptors for MBFV SL and TBFV Y-SL xrRNA structures were further used for searching in the 3′UTRs of genomes from the cISFV group, for which no general xrRNA motif had been previously described. Interestingly, in some of the cISFV 3′UTRs, the conserved structures were detected by the MBFV SL descriptor, in some by TBFV Y-SL one, and in some 3′UTRs, the same structure was identified by both descriptor types. Although this heterogeneous cISFV clade is characterized by considerable sequence diversity, with host-specific subgroups (Blitvich and Firth, 2015; Colmant *et al.*, 2017), the similarities in the detected structures allowed us to identify the common xrRNA motif in the main subgroups of viruses infecting different mosquito species: *Aedes*, *Culex* and *Anopheles*. After realignment of repeated structures in cISFV 3′UTRs (Fig. 5a), two cISFV consensus xrRNA descriptors with different size ranges of their structural elements were designed and included into the database (Fig. 5b). Similar to splitting between two TBFV Y-SL descriptors, the two cISFV variants differed in the sizes of the S3-P1′ loop of the general flavivirus xrRNA model (Akiyama *et al.*, 2016; MacFadden *et al.*, 2018) (Fig. 5c). The only 3′UTR of the cISFV group, where we could not reliably identify this motif, is the one of *Anopheles*-associated Karumba virus. Although some parts of consensus structure could be found by manual inspection of the sequence, a complete consensus-matching topology was not detected.

The cISFV consensus xrRNA motif in *Aedes*, *Culex* and *Anopheles* flaviviruses extends previous predictions of such structures in 3′UTRs of *Aedes*-associated cell fusing agent virus, Aedes flavivirus and Kamiti River virus (MacFadden *et al.*, 2018). The numbers of sites matching this motif were diverse in different cISFV 3′UTRs: in some viruses, only one structure was found, while 2, 3, 4 or even 7 sites could be detected in others (Fig. 5a). In these cases, the sequences in the duplicated structures are not identical, with a number of covariations supporting the conserved topology.

In general, it was noted that the search algorithm frequently yielded numerous hits corresponding to the region of a single phylogenetically conserved structure due to variation of stem and loop sizes allowed by a corresponding descriptor. Sorting of hits according to their free energy values allowed to identify correct structures which were among the first in the list (Supplementary Table S2). In the majority of cases, the very first structure in a cluster was the correct one, and more than 90% of correct structures were among the top 5 structures in their clusters (Supplementary Table S3). Thus the inspection of the conserved features in a few most stable structures from a cluster of matches yielded by the algorithm is mostly sufficient for the identification of the searched functional flavivirus 3′UTR element. Minor differences between top matches in the same cluster sometimes did not allow to resolve the details of phylogenetically conserved topology, yielding potential alternatives. Nevertheless, the functional xrRNA SL structures in the majority of viruses turned out to be locally optimal in terms of stacking free energy (Supplementary Table S3). Furthermore, the phylogenetically supported structures with suboptimal free energy values were mostly only marginally less stable than the rank 1 matches (Supplementary Table S2).

The ranges of stacking free energy values of representative flavivirus RNA 3′UTR structures (Supplementary Table S3), provided the basis for filtering out false-positive matches corresponding to less stable conformations. On the other hand, to prevent missing potential functional structures in future searches with newly sequenced flaviviruses, the opportunity to see the suspicious matches with relatively high free energies was left in the database application. In general, for all used descriptors (Figs 4, 5b), the matches with free energies above −20 kcal/mol are most likely to be false positives.

Of course, in case of duplicated structures, a number of hits corresponding to one structure could precede the correct structure of the repeat. However, restricting the number of hits per region (see Section 2) allows to scroll down through the list of hits to retrieve the repeated structure. In a number of cases, a conserved structure could yield the hits in the searches with different descriptors due to unavoidable overlaps between them. For instance, different DB descriptors frequently yielded the hits at the same sites. As mentioned above, the consensa defined by descriptors for xrRNA SL structures also overlapped.

Conserved structures were not found in Entebbe bat virus (NKV group), apparently because of incompleteness and/or deletions in the available 3′UTR sequence (NC_008718). In the duplicated parts of 3′UTRs of Tyuleniy and Kama viruses from the TBFV group, potential conserved pseudoknot-containing structures could be predicted (not shown). However, these structures were not similar to the structures conserved in other flaviviruses. Therefore, in the absence of any data on the functional implication, we did not include this motif into the database.

The designed descriptors detected potential xrRNA structures in the 3′UTRs of several flaviviruses distantly related to the main ecological groups (not shown). For instance, two structures matching the cISFV consensus xrRNA motif were detected in Tamana bat virus, one of them being also obtained with the TBFV Y-SL descriptor. Putative xrRNA structures were revealed by the cISFV or MBFV SL descriptors in some of the viruses isolated from marine hosts (Parry and Asgari, 2019), such as Gammarus chevreuxi flavivirus, Crangon crangon flavivirus and Southern pygmy squid flavivirus, but homologues of these structures were not found in related viruses. Apparently, the structures in these viruses are more diverse than those in the main flavivirus groups. While the development of essentially new structural models is not within the scope of this manuscript, in the future it will be easy to update the database using new consensa.

The running times on flaviviral RNAs were variable. In the current database implementation, the majority of searches yielded results in seconds. However, in some specific cases, the scanning of a flavivirus 3′UTR took longer. Obviously, the speed depended on the descriptor type, mainly due to combinatorial complexity determined by allowed length variabilities of stems and loops. The cISFV SL descriptor turned out to be the slowest. While the MBFV or TBFV SL jobs could be completed in few seconds, the searches for cISFV SL structures could take minutes, e.g. with CPU times 192 and 250 s for Sabethes flavivirus and Culex flavivirus, respectively. The computation times were not directly proportional to the number of repeated SL structures. The Culex flavivirus 3′UTR contains four SL repeats while the Sabethes flavivivirus only one (Fig. 5a), what was not reflected in the difference between the computation times. A similar search in Nienokoue virus 3′UTR with seven repeats took only 141 s of CPU time, and the detection of the single SL structure in Culiseta flavivirus 3′UTR took just 15 s of CPU. Apparently, it was the number of candidate regions (Fig. 3d) to be analyzed in detail by the algorithm that significantly contributed to the computation times.

An adequate comparison of the algorithm performance with available similar algorithms was only possible for RNABOB program (S. Eddy, unpublished; eddylab.org), which is an implementation of the RNAMOT algorithm (Gautheret *et al.* 1990). Similar to our algorithm, RNABOB does not require sequence alignment, is based on sequence descriptors and has been successfully applied for the search of complex pseudoknot structures (Riccitelli and Lupták, 2010). Applied to flavivirus pseudoknotted structures, characterized by rather lenient descriptors, our algorithm outperforms the RNABOB (Supplementary Table S4). Even when corrected for potential maximal 8-fold acceleration due to parallel computation on 8 cores in the current implementation, not possible for RNABOB, the flavivirus database searches were executed faster in a number of cases. Moreover, in the overwhelming majority of flavivirus RNA 3′UTR sequences the RNABOB algorithm identified only the location of a structure satisfying the used descriptor, yielding a single match that incorrectly predicted some parts of the functional conformation, whereas our algorithm exhaustively processed clusters of low-energy descriptor-matching structures to find optimal ones (Supplementary Table S4). Obviously, such optimizations of predictions did require additional time, while being absolutely necessary for finding functional structures. Comparisons to the algorithms for pseudoknotted structure searching based on sequence alignments (Cai *et al.*, 2003; Gautheret and Lambert, 2001; Huang *et al.*, 2008; Lambert *et al.*, 2004) do not make sense because alignments of flavivirus 3′UTR sequences are inconsistent with homology of structural elements due to their diversity and duplications.

In summary, the designed descriptors allowed us to build a database of conserved functional motifs in flavivirus 3′UTRs. The database allows a user to determine the location of important RNA structures and the sequences of potential subgenomic RNAs using 3′UTR sequences as the input.

## 4. Discussion

The results of testing the search algorithm on a number of flavivirus genomic sequences showed that functional flavivirus RNA 3′UTR structures can be identified by threading of base-pairing topology descriptors through target sequences. The number of constraints in conserved structures is sufficient to define a few descriptors that cover their diversity. The advantage of the search with minimal or no sequence requirements is the identification of all sequences that match the defined topology variants irrespectively of sequence similarity to previously known cases. This allows to overcome some of the difficulties in the search for structured RNA motifs with considerable sequence and structure variability, which are especially serious in pseudoknotted structures (Gautheret and Lambert, 2001; Menzel *et al*., 2009).

In general, RNA motif search approaches using manual construction of structural descriptors may suffer from deficiencies in generalization abilities of ‘experts’ based on available data (Menzel *et al.*, 2009; Mosig *et al*., 2009). Notwithstanding this inevitable problem, in cases of extensive structural information such an approach can be more practical than an automatic extraction of structural patterns, e.g. by machine learning algorithms. Strongly conserved pseudoknot and triple base interactions in flavivirus RNA structures, derived from functional and structural data (Akiyama *et al.*, 2016; Chapman *et al.*, 2014; MacFadden *et al.*, 2018; Villordo *et al.*, 2016) rather than from a generalization hypothesis, are the reasons to advocate the construction of a descriptor database as suggested here.

Previously, a descriptor-based approach has successfully been applied for the search for HDV-like ribozyme structures that contain double pseudoknot motifs (Riccitelli and Lupták, 2010; Webb *et al*., 2009). Apart from structural constraints, the HDV-like ribozyme descriptors included a few specified nucleotides in the motifs. In case of flavivirus RNA structures, we decided to avoid implementing such sequence constraints as long as topological ones are sufficient, so as not to influence the odds to discover new sequences with the same structural topologies.

In principle, it is possible to make descriptors even more universal, for instance, to use broader ranges of stem and loop sizes to capture potential structures slightly deviating from the previously known ones. Furthermore, it is possible to design a general descriptor for Xrn1-resistant structures, suitable for detection of both MBFV-specific SL and TBFV-specific Y-SL structures in MBFV, TBFV, NKV and cISFV RNA sequences. However, we found that this damaged the search algorithm performance due to considerable increase of computation time and false discovery rate. Therefore, the database was built with more specific descriptors, currently 11 are included (Figs 4, 5b).

Of course, the absence of sequence constraints in the descriptors increases the probability of false-positive results in the structure searches. However, this probability is significantly decreased by filtering out unrealistic predictions with higher folding energies. The essential feature of structure-guided searches for homologous orthodox RNA secondary structures are thermodynamic stability estimates (Reinkensmeier and Giegerich, 2015; Will *et al*., 2013). Similarly, we observed that when searching for flavivirus pseudoknotted structures, filtering with stacking free energies was essential to distinguish functional structures from false-positive results. Since realistic estimates of pseudoknot loop free energies are limited to more simple pseudoknots (e.g. Andronescu *et al*., 2010; Gultyaev *et al*., 1999), we restricted our simplified energy model to thermodynamics of stems. This showed that stacking energies in flavivirus RNA structures were considerably lower than in random descriptor-matching sequences, and stacking-based filtering was mostly sufficient for correct structure identification. Furthermore, unlikely loop sizes were mostly filtered out by descriptor design on the basis of previously determined structures.

Apparently, the descriptor-based searches of homologous RNA structures and database construction can complement the construction of RNA families by CM approach when diversity of sequences and complexity of structures are relatively high. For instance, sequence searches in the Rfam database (https://rfam.org; Kalvari *et al.*, 2018) failed to identify SL and DB structures in reference MBFV genomes of dengue 2 virus (accession NC_001474) and yellow fever virus (accession NC_002031), respectively, and yielded no hits in MBFV-related Yokose virus (accession NC_005039), despite the presence of MBFV SL and DB families in the database (families RF02549 and RF00525). The CM designs for subgroup-specific xrRNA motifs within the cISFV clade could not identify the homologues in other subgroups (Ochsenreiter *et al.*, 2019). For instance, the CMs constructed on the basis of xrRNA structures in *Aedes*-associated cell fusing agent virus, Aedes flavivirus and Kamiti River virus 3′UTRs did not yield matches in genomes of *Culex*-associated viruses and even in those from *Aedes*-associated viruses that were not used for this CM design (Ochsenreiter *et al.*, 2019). Furthermore, the alignment of repeated sequences in a subgroup of *Culex*-associated viruses results in a CM model that captures only parts of the conserved xrRNA motif, erroneously predicting orthodox secondary structure elements instead of important tertiary interactions because of the pseudoknot-free CM approximation. Such a model does not detect similar structures in other *Culex*-associated viruses (see Figure 4 of Ochsenreiter *et al.*, 2019). As shown here (Fig. 5, Supplementary Table S2), it is possible to construct unified structure descriptors for searches of members of these structural RNA families in more distant viruses, detecting the same motifs in all of them. On the other hand, a sequence-independent descriptor of this type for less constrained orthodox secondary structures of the 3′SL at the very 3′end of flaviviviral genomes (Fig. 1) would result in long computations and high false discovery rates, while CM patterns identify this motif more reliably, in particular, when split into subfamilies (Ochsenreiter *et al.*, 2019).

It should be noted that we limited the database contents to well-established pseudoknotted structural motifs in flavivirus RNA 3′UTRs. Some flaviviruses may have different RNA structures which could have the same or different functions. Upon elucidation of new functional structures, new descriptors can be readily added to the database.

The database of flavivirus RNA structures can be helpful in both fundamental research and applied analysis of flaviviruses such as diagnostics, genotyping, mutational analysis, vaccine design etc. For instance, in a rational design of safe and efficient vaccines deletion or disruption of flavivirus 3′UTR structures could lead to an attenuated virus while preserving the desired antigenicity (Durbin *et al*., 2001; Mandl *et al*., 1998; Proutski *et al*., 1997; Shan *et al*., 2017). Finally, the database is not only a compilation of known RNA structures, it will hopefully provide an efficient tool for the identification of functional RNA structures in newly discovered viruses and/or isolates.

## Supplementary Material

btaa759_Supplementary_Data
