# Supplementary material for: A database of flavivirus RNA structures with a search algorithm for pseudoknots and triple base interactions
Source: Bioinformatics. 2020 Aug 31;37(7):956–62. doi: 10.1093/bioinformatics/btaa759 (PMC8128465; doi:10.1093/bioinformatics/btaa759)
Supplement: btaa759_Supplementary_Data [file btaa759_supplementary_data.pdf]

Supplementary data for the article

A database of flavivirus RNA structures with a search algorithm for pseudoknots and triple base interactions

by A. Zammit, L. Helwerda, R.C.L. Olsthoorn, F.J. Verbeek and A.P. Gultyaev

Supplementary Table S1. Nucleotide positions of flavivirus 3'UTR RNA structural elements used for design of structure descriptors.

virus	accession	ORF 3'end	MBFV SL	TBFV Y-SL	DB1 (without PK)	DB2 + PK	TBFV 5'GC-SL	TBFV 3'GC-SL	TBFV AU-SL
**Mosquito-borne flaviviruses (MBFV)**									
Japanese encephalitis virus	NC_001437	10394	10458-10517<br>10618-10668		10704-10774	10785-10866			
Usutu virus	NC_006551	10401	10545-10604<br>10704-10751		10787-10858	10865-10955			
Murray Valley encephalitis virus	NC_000943	10400	10494-10554<br>10656-10704		10740-10809	10817-10904			
Alfuy virus	AY898809	10400	10438-10495<br>10598-10645		10681-10750	10759-10853			
West Nile virus lineage 1	NC_009942	10398	10505-10565<br>10665-10719		10760-10826	10836-10925			
Kunjin virus	L24512	3	103-163<br>263-317		357-425	434-523			
Koutango virus	L48980	239	308-367<br>468-521						
Cacipacore virus	LN849009	10284	10492-10547		10719-10788				
Saint Louis encephalitis virus	NC_007580	10391	10430-10489<br>10588-10641		10677-10742	10748-10836			
Ilheus virus	NC_009028	10367	10395-10452		10488-10554	10561-10651			
T'Ho virus	NC_034151	10381	10573-10629		10664-10729	10739-10830			
Ntaya virus	NC_018705	10378	10492-10551<br>10651-10707		10744-10810	10819-10907			
Bagaza virus	NC_012534	10375	10493-10554<br>10655-10711			10737-10825			
Tembusu virus	NC_015843	10372	10468-10527<br>10626-10683		10720-10788	10796-10886			
Kokobera virus	NC_009029	10316	10351-10401<br>10513-10568		10605-10672	10684-10774			
Iguape virus	AY632538	10355	10386-10439<br>10554-10607		10645-10706	10717-10815			

virus	accession	ORF 3'end	MBFV SL	TBFV Y-SL	DB1 (without PK)	DB2 + PK	TBFV 5'GC-SL	TBFV 3'GC-SL	TBFV AU-SL
Bussuquara virus	NC_009026	10394	10424-10477			10617-10713			
Stratford virus	KM225263	10327	10366-10416						
New Mapoon virus	KC788512	10318	10356-10405<br>10516-10572		10606-10676	10682-10765			
Zika virus	NC_012532	10366	10383-10437<br>10467-10518			10603-10688			
Kedougou virus	NC_012533	10333	10401-10457			10529-10617			
Dengue virus 1	NC_001477	10273	10324-10375<br>10397-10444			10467-10549<br>10551-10633			
Dengue virus 2	NC_001474	10272	10303-10355<br>10377-10428			10453-10534<br>10540-10621			
Dengue virus 3	NC_001475	10267	10295-10346<br>10370-10418			10439-10522<br>10524-10605			
Dengue virus 4	NC_002640	10265	10296-10345			10376-10462<br>10467-10547			
Yellow fever virus	NC_002031	10354	10536-10602			10663-10749			
Sepik virus	NC_008719	10334	10481-10538			10597-10682			
Wesselsbron virus	NC_012735	10336	10497-10554			10611-10701			
Uganda S virus	AY326409	3	48-102						
Banzi virus	AY326407	3	152-207						
Fitzroy River virus	KM361634	10335	10496-10553			10609-10695			
Paraiso Escondido virus	NC_027999	10445	10459-10512<br>10541-10588						
**MBFV-related viruses with no known vector (NKV)**									
Yokose virus	NC_005039	10428	10457-10515			10635-10743			
**MBFV-related insect-specific flaviviruses (ISFV)**									
Chaoyang virus	NC_017086	10407	10447-10499			10538-10620			
Lammi virus	FJ606789	10401	10439-10491			10532-10614			
Donggang virus	NC_016997	10448	10474-10533			10589-10678			
Marisma mosquito virus	MF139576	10472	10527-10586			10643-10732			

virus	accession	ORF 3'end	MBFV SL	TBFV Y-SL	DB1 (without PK)	DB2 + PK	TBFV 5'GC-SL	TBFV 3'GC-SL	TBFV AU-SL
Nounane virus	NC_033715	10408	10473-10527						
Nanay virus	MF139575	10405	10470-10523			10603-10688			
Barkedji virus	MG214905	10358	10430-10490						
Nhumirim virus	NC_024017	10440	10522-10586			10688-10774			
**Tick-borne flaviviruses**									
Tick-borne encephalitis virus, strain Neudoerfl	NC_001672	10377		10697-10771<br>10830-10910			10773-10821	10910-10976	10977-11032
Tick-borne encephalitis virus, clone Hypr_IC	KP716974	10377		10654-10728<br>10791-10871			10729-10782	10872-10938	10939-10994
Louping ill virus	NC_001809	10374		10430-10504<br>10562-10640			10505-10550	10642-10705	10706-10762
Tick-borne encephalitis virus, strain Vasilchenko	L40361	10377		10487-10561<br>10619-10697			10562-10608	10697-10762	10763-10819
Tick-borne encephalitis virus, strain Sofjin	JX498940	10376		10455-10529<br>10584-10664			10530-10574	10665-10729	10730-10786
Spanish goat encephalitis virus	NC_027709	10377		10423-10497<br>10562-10639			10499-10551	10640-10705	10706-10762
Omsk hemorrhagic fever virus	AY193805	10377		10479-10557			10421-10464	10559-10622	10623-10679
Langat virus	NC_003690	10375		10505-10583<br>10634-10715			10584-10625	10716-10778	10779-10835
Alkhumra hemorrhagic fever virus	AF331718	10365		10457-10531			10402-10447	10534-10589	10590-10647
Kyasanur forest disease virus	HM055369	10382		10473-10547			10418-10463	10552-10605	10606-10663
Powassan virus	NC_003687	10359		10404-10485<br>10537-10609			10486-10529	10614-10673	10674-10730
Deer tick virus	AF311056	10341		10386-10465<br>10518-10590			10466-10510	10595-10654	10655-10711
Karshi virus	AY863002	10272		10291-10366<br>10427-10506			10370-10413	10508-10561	10562-10619
Negishi virus	KT224355	10339		10394-10469			10471-10522		
**TBFV-related viruses with no known vector (NKV)**									
Modoc virus	NC_003635	10234		10264-10340	10354-10429				10444-10502

virus	accession	ORF 3'end	MBFV SL	TBFV Y-SL	DB1 (without PK)	DB2 + PK	TBFV 5'GC-SL	TBFV 3'GC-SL	TBFV AU-SL
Rio Bravo virus	JQ582840	10256		10320-10393	10463-10536				10589-10642
Montana myotis leukoencephalitis virus	AJ299445	10233		10290-10362	10425-10486				10534-10589
Apoi virus	AF452050	<1		22-105<br>208-260	263-309				392-451
**Classic insect-specific flaviviruses (cISFV)**									
Cell fusing agent virus	NC_001564	10129	10176-10224<br>10263-10310						
Aedes flavivirus	NC_012932	10122	10499-10547<br>10637-10684						
Kamiti River virus	NC_005064	10170	10361-10408<br>10957-11004						

Supplementary Table S2. Locally optimal and suboptimal predictions of phylogenetically conserved structures. Ranks of structural elements, predicted by the algorihm in clusters of descriptor-matching structures in specific regions, arranged in ascending order of stacking free energies ($\Delta G_{stacking}$). For the structures with suboptimal free energies, the differences compared to the free energies of rank 1 matches are shown ($\Delta\Delta G_{stacking}$).

virus	accession	ORF 3'end	structure	position	structure rank in the cluster	$\Delta G_{stacking}$ (kcal/mol)	$\Delta\Delta G_{stacking}$ (kcal/mol)
**Mosquito-borne flaviviruses (MBFV)**							
Japanese encephalitis virus	NC_001437	10394	MBFV SL	10458-10517	2	-37.1	0.7
			MBFV SL	10618-10668	1	-37.5	
			DB1 (without PK)	10704-10774	1	-35.8	
			DB2 +PK	10785-10866	2	-40.7	0.3
Usutu virus	NC_006551	10401	MBFV SL	10545--10604	1	-42.5	
			MBFV SL	10704-10751	1	-32.5	
			DB1 (without PK)	10787-10858	1	-28.3	
			DB2 + PK	10865-10955	4	-43.9	0.3
Murray Valley encephalitis virus	NC_000943	10400	MBFV SL	10494-10554	4	-44.4	3.3
			MBFV SL	10656-10704	1	-30.0	
			DB1 (without PK)	10740-10809	1	-32.8	
			DB2 + PK	10817-10904	2	-43.0	0.3
Alfuy virus	AY898809	10400	MBFV SL	10438-10495	1	-39.3	
			MBFV SL	10598-10645	1	-29.5	
			DB1 (without PK)	10681-10750	1	-35.8	
			DB2 + PK	10759-10853	2	-45.7	0.3
West Nile virus lineage 1	NC_009942	10398	MBFV SL	10505-10565	1	-43.5	
			MBFV SL	10665-10719	1	-35.2	
			DB1 (without PK)	10760-10826	1	-28.3	

virus	accession	ORF 3'end	structure	position	structure rank in the cluster	$\Delta G_{stacking}$ (kcal/mol)	$\Delta\Delta G_{stacking}$ (kcal/mol)
			DB2 + PK	10836-10925	2	-43.2	0.3
Kunjin virus	L24512	3	MBFV SL	103-163	1	-39.3	
			MBFV SL	263-317	1	-30.5	
			DB1 (without PK)	357-425	2	-29.2	0
			DB2 + PK	434-523	2	-43.2	0.3
Koutango virus	L48980	239	MBFV SL	308-367	1	-39.3	
			MBFV SL	468-521	1	-30.9	
Cacipacore virus	LN849009	10284	MBFV SL	10492-10547	1	-38.0	
			DB1 (without PK)	10719-10788	1	-30.9	
Saint Louis encephalitis virus	NC_007580	10391	MBFV SL	10430-10489	1	-36.1	
			MBFV SL	10588-10641	1	-29.9	
			DB1 (without PK)	10677-10742	1	-25.4	
			DB2 + PK	10748-10836	2	-46.8	0.2
Ilheus virus	NC_009028	10367	MBFV SL	10395-10452	1	-37.4	
			DB1 (without PK)	10488-10554	1	-26.5	
			DB2 + PK	10561-10651	4	-43.5	1.1
T'Ho virus	NC_034151	10381	MBFV SL	10573-10629	1	-37.4	
			DB1 (without PK)	10664-10729	1	-28.4	
			DB2 + PK	10739-10830	2	-46.5	0.3
Ntaya virus	NC_018705	10378	MBFV SL	10492-10551	1	-39.9	
			MBFV SL	10651-10707	2	-31.6	0.2
			DB1 (without PK)	10744-10810	1	-29.6	
			DB2 + PK	10819-10907	2	-39.0	0.3

virus	accession	ORF 3'end	structure	position	structure rank in the cluster	$\Delta G_{stacking}$ (kcal/mol)	$\Delta\Delta G_{stacking}$ (kcal/mol)
Bagaza virus	NC_012534	10375	MBFV SL	10493-10554	1	-43.0	
			MBFV SL	10655-10711	2	-32.1	0.2
			DB2 + PK	10737-10825	1	-42.3	
Tembusu virus	NC_015843	10372	MBFV SL	10468-10527	1	-39.7	
			MBFV SL	10626-10683	2	-32.6	0.2
			DB1 (without PK)	10720-10788	7	-28.1	3.3
			DB2 + PK	10796-10886	2	-43.2	0.3
Kokobera virus	NC_009029	10316	MBFV SL	10351-10401	2	-32.4	0.2
			MBFV SL	10513-10568	1	-33.9	
			DB1 (without PK)	10605-10672	1	-29.3	
			DB2 + PK	10684-10774	2	-42.4	0.7
Iguape virus	AY632538	10355	MBFV SL	10386-10439	1	-36.8	
			MBFV SL	10554-10607	1	-36.2	
			DB1 (without PK)	10645-10706	2	-17.8	0.9
			DB2 + PK	10717-10815	2	-47.6	0.7
Bussuquara virus	NC_009026	10394	MBFV SL	10424-10477	18	-25.4	8.0
			DB2 + PK	10617-10713	2	-32.8	0.4
Bainyik virus	KM225264	10277	MBFV SL	10311-10361	1	-34.9	
			MBFV SL	10473-10528	1	-37.2	
			DB1 (without PK)	10565-10633	1	-26.0	
Torres virus	KM225265	10321	MBFV SL	10351-10402	1	-30.2	
			MBFV SL	10513-10568	1	-30.3	
			DB1 (without PK)	10605-10672	1	-25.4	

virus	accession	ORF 3'end	structure	position	structure rank in the cluster	ΔG$_{stacking}$ (kcal/mol)	ΔΔG$_{stacking}$ (kcal/mol)
Stratford virus	KM225263	10327	MBFV SL	10366-10416	1	-30.3	
New Mapoon virus	KC788512	10318	MBFV SL	10356-10405	3	-28.9	2.2
			MBFV SL	10516-10572	1	-34.8	
			DB1 (without PK)	10606-10676	2	-32.3	0.3
			DB2 + PK	10682-10765	1	-39.7	
Zika virus	NC_012532	10366	MBFV SL	10383-10437	4	-37.1	1.2
			MBFV SL	10467-10518	2	-28.9	
			DB2 + PK	10603-10688	2	-46.2	1.5
Kedougou virus	NC_012533	10333	MBFV SL	10401-10457	1	-38.1	
			DB2 + PK	10529-10617	1	-44.0	
Dengue virus 1	NC_001477	10273	MBFV SL	10324-10375	2	-28.3	2.1
			MBFV SL	10397-10444	1	-33.4	
			DB2 + PK	10467-10549	1	-44.2	
			DB2 + PK	10551-10633	1	-50.7	
Dengue virus 2	NC_001474	10272	MBFV SL	10303-10355	6	-21.6	4.6
			MBFV SL	10377-10428	1	-25.5	
			DB2 + PK	10453-10534	1	-43.2	
			DB2 + PK	10540-10621	1	-45.7	
Dengue virus 3	NC_001475	10267	MBFV SL	10295-10346	6	-23.5	4.6
			MBFV SL	10370-10418	1	-33.0	
			DB2 + PK	10439-10522	1	-46.9	
			DB2 + PK	10524-10605	1	-44.4	
Dengue virus 4	NC_002640	10265	MBFV SL	10296-10345	1	-34.3	
			DB2 + PK	10376-10462	1	-46.3	
			DB2 + PK	10467-10547	1	-47.2	

virus	accession	ORF 3'end	structure	position	structure rank in the cluster	$\Delta G_{stacking}$ (kcal/mol)	$\Delta\Delta G_{stacking}$ (kcal/mol)
Yellow fever virus	NC_002031	10354	MBFV SL	10536-10602	1	-41.4	
			DB2 + PK	10663-10749	1	-47.9	
Sepik virus	NC_008719	10334	MBFV SL	10481-10538	1	-38.9	
			DB2 + PK	10597-10682	1	-50.2	
Wesselsbron virus	NC_012735	10336	MBFV SL	10497-10554	1	-41.2	
			DB2 + PK	10611-10701	1	-45.9	
Uganda S virus	AY326409	3	MBFV SL	48-102	1	-30.4	
Banzi virus	AY326407	3	MBFV SL	152-207	1	-38.2	
Fitzroy River virus	KM361634	10335	MBFV SL	10496-10553	1	-41.2	
			DB2 + PK	10609-10695	1	-45.7	
Paraiso Escondido virus	NC_027999	10445	MBFV SL	10459-10512	1	-35.0	
			MBFV SL	10541-10588	1	-24.0	
**MBFV-related viruses with no known vector (NKV)**							
Yokose virus	NC_005039	10428	MBFV SL	10457-10515	1	-36.8	
			DB2 + PK	10635-10473	1	-55.1	
**MBFV-related insect-specific flaviviruses (ISFV)**							
Chaoyang virus	NC_017086	10407	MBFV SL	10447-10499	2	-32.0	2.1
			DB2 + PK	10538-10620	1	-50.8	
Lammi virus	FJ606789	10401	MBFV SL	10439-10491	1	-33.4	
			DB2 + PK	10532-10614	1	-48.7	
Donggang virus	NC_016997	10448	MBFV SL	10474-10533	7	-36.5	5.5
			DB2 + PK	10589-10678	1	-54.2	
Marisma mosquito virus	MF139576	10472	MBFV SL	10527-10586	1	-40.4	
			DB2 + PK	10643-10732	1	-49.4	
Nounane virus	NC_033715	10408	MBFV SL	10473-10527	3	-30.9	2.1
Nanay virus	MF139575	10405	MBFV SL	10470-10523	1	-35.2	

virus	accession	ORF 3'end	structure	position	structure rank in the cluster	$\Delta G_{stacking}$ (kcal/mol)	$\Delta\Delta G_{stacking}$ (kcal/mol)
			DB2 + PK	10603-10688	1	-42.5	
Barkedji virus	MG214905	10358	MBFV SL	10430-10490	4	-31.8	1.4
Nhumirim virus	NC_024017	10440	MBFV SL	10522-10586	1	-42.2	
			DB2 + PK	10688-10774	1	-44.4	
**Tick-borne flaviviruses**							
Tick-borne encephalitis virus, strain Neudoerfl	NC_001672	10377	TBFV Y-SL	10697-10771	1	-34.9	
			TBFV Y-SL	10830-10910	1	-37.4	
			TBFV 5'GC-SL	10773-10821	1	-40.4	
			TBFV 3'GC-SL	10910-10976	1	-44.1	
			TBFV AU-SL	10977-11032	1	-34.6	
Tick-borne encephalitis virus, clone Hypr_IC	KP716974	10377	TBFV Y-SL	10654-10728	1	-34.9	
			TBFV Y-SL	10791-10871	1	-37.4	
			TBFV 5'GC-SL	10729-10782	1	-44.6	
			TBFV 3'GC-SL	10872-10938	1	-40.4	
			TBFV AU-SL	10939-10994	1	-34.6	
Louping ill virus	NC_001809	10374	TBFV Y-SL	10430-10504	1	-33.0	
			TBFV Y-SL	10562-10640	1	-32.6	
			TBFV 5'GC-SL	10505-10550	1	-33.7	
			TBFV 3'GC-SL	10642-10705	1	-36.8	
			TBFV AU-SL	10706-10762	1	-39.0	
Tick-borne encephalitis virus, strain Vasilchenko	L40361	10377	TBFV Y-SL	10487-10561	1	-34.9	
			TBFV Y-SL	10619-10697	1	-35.2	
			TBFV 5'GC-SL	10562-10608	2	-35.9	0.6
			TBFV 3'GC-SL	10697-10762	1	-45.1	
			TBFV AU-SL	10763-10819	1	-33.3	
Tick-borne encephalitis virus, strain Sofjin	JX498940	10376	TBFV Y-SL	10455-10529	1	-34.9	

virus	accession	ORF 3'end	structure	position	structure rank in the cluster	$\Delta G_{stacking}$ (kcal/mol)	$\Delta\Delta G_{stacking}$ (kcal/mol)
			TBFV Y-SL	10584-10664	1	-34.9	
			TBFV 5'GC-SL	10530-10574	1	-30.9	
			TBFV 3'GC-SL	10665-10729	1	-44.2	
			TBFV AU-SL	10730-10786	1	-37.7	
Spanish goat encephalitis virus	NC_027709	10377	TBFV Y-SL	10423-10497	1	-34.9	
			TBFV Y-SL	10562-10639	1	-35.5	
			TBFV 5'GC-SL	10499-10551	1	-40.1	
			TBFV 3'GC-SL	10640-10705	1	-44.7	
			TBFV AU-SL	10706-10762	1	-37.9	
Omsk hemorrhagic fever virus	AY193805	10377	TBFV Y-SL	10479-10557	1	-35.2	
			TBFV 5'GC-SL	10421-10464	1	-29.3	
			TBFV 3'GC-SL	10559-10622	1	-41.8	
			TBFV AU-SL	10623-10679	1	-37.2	
Langat virus	NC_003690	10375	TBFV-Y-SL	10505-10583	1	-34.4	
			TBFV Y-SL	10634-10715	1	-38.0	
			TBFV 5'GC-SL	10584-10625	1	-29.3	
			TBFV 3'GC-SL	10716-10778	1	-45.8	
			TBFV AU-SL	10779-10835	1	-37.9	
Alkhumra hemorrhagic fever virus	AF331718	10365	TBFV Y-SL	10457-10531	4	-36.2	2.1
			TBFV 5'GC-SL	10402-10447	1	-30.6	
			TBFV 3'GC-SL	10534-10589	1	-41.4	
			TBFV AU-SL	10590-10647	1	-41.0	
Kyasanur forest disease virus	HM055369	10382	TBFV Y-SL	10473-10547	1	-38.3	
			TBFV 5'GC-SL	10418-10463	1	-30.2	
			TBFV 3'GC-SL	10552-10605	1	-37.9	
			TBFV AU-SL	10606-10663	1	-41.0	

virus	accession	ORF 3'end	structure	position	structure rank in the cluster	$\Delta G_{stacking}$ (kcal/mol)	$\Delta\Delta G_{stacking}$ (kcal/mol)
Powassan virus	NC_003687	10359	TBFV Y-SL	10404-10485	3	-35.5	3.3
			TBFV Y-SL	10537-10609	4	-29.4	2.1
			TBFV 5'GC-SL	10486-10529	1	-30.8	
			TBFV 3'GC-SL	10614-10673	1	-38.8	
			TBFV AU-SL	10674-10730	1	-36.5	
Deer tick virus	AF311056	10341	TBFV Y-SL	10386-10465	1	-32.6	
			TBFV Y-SL	10518-10590	1	-38.8	
			TBFV 5'GC-SL	10466-10510	1	-29.6	
			TBFV 3'GC-SL	10595-10654	1	-38.0	
			TBFV AU-AL	10655-10711	1	-38.6	
Karshi virus	AY863002	10272	TBFV Y-SL	10291-10366	4	-33.2	3.3
			TBFV Y-SL	10427-10506	1	-32.9	
			TBFV 5'GC-SL	10370-10413	1	-30.0	
			TBFV 3'GC-SL	10508-10561	1	-32.0	
			TBFV AU-SL	10562-10619	1	-41.6	
Negishi virus	KT224355	10339	TBFV Y-SL	10394-10469	1	-39.6	
			TBFV 5'GC-SL	10471-10522	1	-40.4	
**TBFV-related viruses with no known vector (NKV)**							
Modoc virus	NC_003635	10234	TBFV Y-SL	10264-10340	1	-39.8	
			DB1 (without PK)	10354-10429	1	-37.8	
			TBFV AU-SL	10444-10502	1	-38.6	
Rio Bravo virus	JQ582840	10256	TBFV Y-SL	10320-10393	5	-29.9	3.3
			DB1 (without PK)	10463-10536	1	-34.8	
			TBFV AU-SL	10589-10642	1	-35.2	
Montana myotis leukoencephalitis virus	AJ299445	10233	TBFV Y-SL	10290-10362	10	-26.1	4.2
			DB1 (without PK)	10425-10486	1	-28.7	

virus	accession	ORF 3'end	structure	position	structure rank in the cluster	$\Delta G_{stacking}$ (kcal/mol)	$\Delta\Delta G_{stacking}$ (kcal/mol)
			TBFV AU-SL	10534-10589	1	-38.1	
Apoi virus	AF452050	<1	TBFV Y-SL	22-105	1	-38.0	
			TBFV Y-SL	208-260	1	-32.3	
			DB1 (without PK)	263-309	1	-24.5	
			TBFV AU-SL	392-451	1	-35.2	
**Classic insect-specific flaviviruses (cISFV)**							
Cell fusing agent virus	NC_001564	10129	cISFV SL	10176-10224	1	-30.3	
			cISFV SL	10263-10310	1	-29.7	
Aedes flavivirus	NC_012932	10122	cISFV SL	10499-10547	10	-28.1	2.4
			cISFV SL	10637-10684	1	-28.4	
Kamiti River virus	NC_005064	10170	cISFV SL	10361-10408	1	-28.9	
			cISFV SL	10957-11004	1	-28.9	
Parramatta River virus	KT192549	10264	cISFV SL	10321-10371	1	-32.6	
			cISFV SL	10446-10493	2	-30.3	0.9
			cISFV SL	10543-10594	2	-29.6	0.1
			cISFV SL	10663-10722	2	-38.2	0.1
Ochlerotatus caspius flavivirus	NC_034242	10222	cISFV SL	10282-10330	1	-24.9	
Xishuangbanna aedes flavivirus	NC_034017	10335	cISFV SL	10366-10437	1	-39.8	
			cISFV SL	10749-10805	2	-30.2	0
Menghai flavivirus	NC_034204	10354	cISFV SL	10387-10457	1	-44.7	
Culex flavivirus	NC_008604	10183	cISFV SL	10249-10302	1	-28.1	
			cISFV SL	10368-10421	1	-32.5	
			cISFV SL	10481-10529	21	-29.7	5.3
			cISFV SL	10601-10650	1	-27.3	
Quang Binh virus	NC_012671	10192	cISFV SL	10248-10305	1	-32.0	
			cISFV SL	10347-10405	1	-36.1	

virus	accession	ORF 3'end	structure	position	structure rank in the cluster	$\Delta G_{stacking}$ (kcal/mol)	$\Delta\Delta G_{stacking}$ (kcal/mol)
			cISFV SL	10493-10553	8	-35.2	2.1
			cISFV SL	10623-10676	1	-38.3	
Culex theileri flavivirus	HE574574	10124	cISFV SL	10181-10238	1	-30.2	
			cISFV SL	10264-10343	1	-26.7	
			cISFV SL	10409-10464	1	-37.4	
Nienokoue virus	NC_024299	10169	cISFV SL	10214-10277	1	-42.5	
			cISFV SL	10352-10415	1	-41.0	
			cISFV SL	10432-10483	1	-32.2	
			cISFV SL	10504-10558	1	-29.4	
			cISFV SL	10564-10611	1	-25.8	
			cISFV SL	10640-10689	1	-26.5	
			cISFV SL	10750-10820	1	-32.1	
Mercadeo virus	KP688057	10300	cISFV SL	10591-10643	1	-30.3	
			cISFV SL	10814-10867	3	-25.2	1.6
Calbertado virus	KX669688	10202	cISFV SL	10634-10686	4	-27.4	1.6
Sabethes flavivirus	MH899446	10261	cISFV SL	10337-10392	1	-34.2	
Culiseta flavivirus	KT599442	10282	cISFV SL	10685-10738	1	-31.2	
Anopheles flavivirus variant 1	KX148546	10133	cISFV SL	10144-10198	2	-38.4	0.7
Anopheles flavivirus variant 2	KX148547	10086	cISFV SL	10097-10151	2	-38.4	0.7

Supplementary Table S3. The ranges of stacking free energy values of representative flavivirus RNA 3'UTR structures. The lowest, the highest and median values of the structures given in the Supplementary Table S2 are indicated. The numbers of phylogenetically supported structures with high ranks in the corresponding clusters of descriptor-matching structures, sorted by free energies, are also shown.

structure	min($\Delta G_{stacking}$) (kcal/mol)	max($\Delta G_{stacking}$) (kcal/mol)	median($\Delta G_{stacking}$) (kcal/mol)	number of structures	number of structures with rank=1 in the cluster	number of structures with rank≤2 in the cluster	number of structures with rank≤5 in the cluster
MBFV SL	-44.4	-21.6	-35.0	64	47 (73%)	55 (86%)	60 (94%)
TBFV Y-SL	-39.8	-26.1	-34.9	29	23 (79%)	23 (79%)	28 (97%)
cISFV SL	-44.7	-24.9	-30.3	39	28 (72%)	34 (87%)	36 (92%)
DB1 (without PK)	-37.8	-17.8	-28.7	21	17 (81%)	20 (95%)	20 (95%)
DB2 + PK	-55.1	-32.8	-45.7	37	22 (59%)	35 (95%)	37 (100%)
TBFV 5' GC-SL	-44.6	-29.3	-30.9	14	13 (93%)	14 (100%)	14 (100%)
TBFV 3'GC-SL	-45.8	-32.0	-41.4	13	13 (100%)	13 (100%)	13 (100%)
TBFV AU-SL	-41.6	-33.3	-37.9	17	17 (100%)	17 (100%)	17 (100%)

Supplementary Table S4. Comparison of the algorithm performance with the descriptor-based RNABOB algorithm (S. Eddy, unpublished; eddylab.org). The RNABOB searches were executed with descriptors equivalent to those described in this work (Figure 4), designed as described in the RNABOB documentation and in (Gautheret *et al*., 1990; Riccitelli *et al.*, 2010). The comparisons were carried out for flavivirus RNA 3'UTR structures without triple interactions, because these elements could not be taken into acount by RNABOB algorithm. Observed calculation times included initial sequence pre-processing steps, in addition to actual calculations. In cases when the RNABOB hits were present in the clusters of structures yielded by the flavivirus database searches, their ranks are given. The differences in stacking free energies ($\Delta G_{stacking}$) between predicted structures by two algorithms are indicated as well ($\Delta\Delta G_{stacking}$).

Structure / virus	Leiden Flavivirus RNA Structure Database search				RNABOB			
	observed calculation time (s)	runtime CPU 8 cores (s)	$\Delta G_{stacking}$ (kcal/mol)	structure rank in the cluster	runtime CPU 1 core (s)	$\Delta G_{stacking}$ (kcal/mol)	structure rank in the cluster	$\Delta\Delta G_{stacking}$ (kcal/mol)
**DB2 + PK:**								
Japanese encephalitis virus	10.3	8.6	-40.7	2	120.6	-25.2	82	15.5
Usutu virus	104.0	90	-43.9	4	171.4	-42.0	7	1.9
Murray Valley encephalitis virus	10.6	12.1	-43.0	2	132.4	-37.8	14	5.2
Alfuy virus	18.2	12.2	-45.7	2	113.3	-41.5	13	4.2
West Nile virus lineage 1	12.5	11.3	-43.2	2	71.4	-36.5	-	6.7
Kunijn virus	11	16.5	-43.2	2	74.7	-36.5	-	6.7
Saint Louis encephalitis virus	19.6	16.7	-46.8	2	79.7	-37.6	91	9.2
Ilheus virus	11.4	14.5	-43.5	4	76.1	-35.3	82	8.2
T'Ho virus	27.3	24.1	-46.5	2	62.7	-34.9	-	11.6
Ntaya virus	12.7	14.9	-39.0	2	66.4	-37.1	3	1.9
Bagaza virus	13.5	9.8	-42.3	1	70.0	-34.9	-	7.4
Tembusu virus	30.8	27.4	-43.2	2	71.0	-34.9	-	8.3
Kokobera virus	10.0	8.6	-42.4	2	121.7	-37.7	29	4.7
Iguape virus	38.3	21.5	-47.6	2	114.2	-36.3	-	11.3
Bussuquara virus	9.8	6.5	-32.8	2	84.3	-27.5	30	5.3
New Mapoon virus	34.5	53	-39.7	1	115.1	-35.7	13	4.0

Structure / virus	Leiden Flavivirus RNA Structure Database search				RNABOB			
	observed calculation time (s)	runtime CPU 8 cores (s)	$\Delta G_{stacking}$ (kcal/mol)	structure rank in the cluster	runtime CPU 1 core (s)	$\Delta G_{stacking}$ (kcal/mol)	structure rank in the cluster	$\Delta\Delta G_{stacking}$ (kcal/mol)
Zika virus	21.6	10.5	-46.2	2	86.4	-41.0	36	5.2
Kedougou virus	8.4	4.4	-44.0	1	90.3	-41.7	6	2.3
Dengue virus 1	14.9	20.8	-44.2<br>-50.7	1<br>1	85.4	-37.5<br>-45.2	-<br>19	6.7<br>5.5
Dengue virus 2	8.75	9.3	-43.2<br>-45.7	1<br>1	103.2	-34.9<br>-25.5	-<br>192	8.3<br>20.2
Dengue virus 3	29.9	26.4	-46.9<br>-44.4	1<br>1	104.3	-33.9<br>-38.9	-	13.0<br>5.5
Dengue virus 4	7.9	6.9	-46.3<br>-47.2	1<br>1	39.4	-41.3<br>-42.3	13<br>12	5.0<br>4.9
Yellow fever virus	0.75	0.04	-47.9	1	0.3	-35.1	35	12.8
Sepik virus	1.18	0.04	-50.2	1	0.3	-35.5	30	14.7
Wesselsbron virus	0.7	0.05	-45.9	1	0.3	-38.2	7	7.7
Fitzroy River virus	0.9	0.04	-45.7	1	0.3	-37.0	9	8.7
Yokose virus	1.0	0.03	-55.1	1	0.3	-47.6	62	7.5
Chaoyang virus	6.0	4.2	-50.8	1	31.5	-38.4	90	12.4
Lammi virus	11.6	8.9	-48.7	1	33.8	-42.0	-	6.7
Donggang virus	12.2	9.1	-54.2	1	39.5	-44.8	-	9.4
Marisma mosquito virus	7.3	3.8	-49.4	1	49.1	-33.9	118	15.5
Nanay virus	17.6	12.5	-42.5	1	39.4	-31.5	-	11.0
Nhumirim virus	37.0	33.5	-44.4	1	53.4	-27.4	-	17.0
**TBFV 5'GC-SL:**								
Tick-borne encephalitis virus, strain Neudoerfl	3.3	0.15	-40.4	1	0.11	-30.4	40	10.0
Tick-borne encephalitis virus, clone Hypr_IC	2.7	0.15	-44.6	1	0.11	-34.9	31	9.7
Louping ill virus	1.7	0.07	-33.7	1	0.08	-27.0	13	6.7

Structure / virus	Leiden Flavivirus RNA Structure Database search				RNABOB			
	observed calculation time (s)	runtime CPU 8 cores (s)	$\Delta G_{stacking}$ (kcal/mol)	structure rank in the cluster	runtime CPU 1 core (s)	$\Delta G_{stacking}$ (kcal/mol)	structure rank in the cluster	$\Delta\Delta G_{stacking}$ (kcal/mol)
Tick-borne encephalitis virus, strain Vasilchenko	1.9	0.08	-35.9	2	0.08	-26.4	17	9.5
Tick-borne encephalitis virus, strain Sofjin	1.8	0.08	-30.9	1	0.08	-28.9	3	2.0
Spanish goat encephalitis virus	1.7	0.07	-40.1	1	0.08	-34.8	10	5.3
Omsk hemorrhagic fever virus	1.9	0.07	-29.3	1	0.06	-20.9	11	8.4
Langat virus	2.0	0.08	-29.3	1	0.09	-29.2	12	0.1
Alkhumra hemorrhagic fever virus	2.5	0.14	-30.6	1	0.05	-21.1	12	9.5
Kyasanur forest disease virus	2.1	0.13	-30.2	1	0.07	-21.1	12	9.1
Powassan virus	2.3	0.11	-30.8	1	0.11	-15.9	18	14.9
Deer tick virus	1.1	0.05	-29.6	1	0.08	-15.3	14	14.3
Karshi virus	1.4	0.08	-30.0	1	0.06	-12.8	73	17.2
Negishi virus	0.8	0.02	-40.4	1	0.04	-33.7	9	6.7
**TBFV 3'GC-SL:**								
Tick-borne encephalitis virus, strain Neudoerfl	0.6	0.08	-44.1	1	0.018	-40.8	2	3.3
Tick-borne encephalitis virus, clone Hypr_IC	1.0	0.005	-40.4	1	0.017	-40.4	1	
Louping ill virus	0.9	0.003	-36.8	1	0.015	-36.8	1	
Tick-borne encephalitis virus, strain Vasilchenko	1.3	0.007	-45.1	1	0.015	-41.8	4	3.3
Tick-borne encephalitis virus, strain Sofjin	1.1	0.003	-44.2	1	0.016	-44.2	1	
Spanish goat encephalitis virus	1.3	0.002	-44.7	1	0.013	-44.7	1	
Omsk hemorrhagic fever virus	1.2	0.004	-41.8	1	0.012	-39.3	2	2.5
Langat virus	1.4	0.008	-45.8	1	0.015	-42.9	2	2.9
Alkhumra hemorrhagic fever virus	1.4	0.004	-41.4	1	0.011	-41.4	1	

Structure / virus	Leiden Flavivirus RNA Structure Database search				RNABOB			
	observed calculation time (s)	runtime CPU 8 cores (s)	$\Delta G_{stacking}$ (kcal/mol)	structure rank in the cluster	runtime CPU 1 core (s)	$\Delta G_{stacking}$ (kcal/mol)	structure rank in the cluster	$\Delta\Delta G_{stacking}$ (kcal/mol)
Kyasanur forest disease virus	1.1	0.003	-37.9	1	0.013	-37.9	1	
Powassan virus	1.3	0.007	-38.8	1	0.018	-32.4	6	6.4
Deer tick virus	0.7	0.005	-38.0	1	0.014	-31.6	6	6.4
Karshi virus	0.9	0.002	-32.0	1	0.012	-32.0	1	
**TBFV AU-SL:**								
Tick-borne encephalitis virus, strain Neudoerfl	1.1	0.01	-34.6	1	0.009	-25.6	29	9.0
Tick-borne encephalitis virus, clone Hypr_IC	0.9	0.007	-34.6	1	0.018	-26.1	29	8.5
Louping ill virus	1.1	0.002	-39.0	1	0.013	-27.0	81	12.0
Tick-borne encephalitis virus, strain Vasilchenko	1.3	0.006	-33.3	1	0.013	-26.8	8	6.5
Tick-borne encephalitis virus, strain Sofjin	1.1	0.015	-37.7	1	0.013	-26.9	102	10.8
Spanish goat encephalitis virus	1.2	0.017	-37.9	1	0.014	-27.0	84	10.9
Omsk hemorrhagic fever virus	1.5	0.012	-37.2	1	0.012	-27.5	76	9.7
Langat virus	1.0	0.01	-37.9	1	0.015	-27.5	79	10.4
Alkhumra hemorrhagic fever virus	1.3	0.05	-41.0	1	0.01	-34.9	51	6.1
Kyasanur forest disease virus	1.0	0.05	-41.0	1	0.012	-34.9	51	6.1
Powassan virus	0.9	0.004	-36.5	1	0.018	-32.1	8	4.4
Deer tick virus	0.8	0.003	-38.6	1	0.013	-34.2	8	4.4
Karshi virus	1.0	0.03	-41.6	1	0.011	-35.1	36	6.5
Modoc virus	0.8	0.006	-38.6	1	0.011	-34.8	6	3.8
Rio Bravo virus	1.0	0.032	-35.2	1	0.013	-30.3	15	4.9
Montana myotis leukoencephalitis virus	0.8	0.005	-38.1	1	0.009	-34.8	5	3.3
Apoi virus	1.2	0.002	-35.2	1	0.014	-32.4	3	2.8

References

Gautheret,D. *et al*. (1990) Pattern searching/alignment with RNA primary and secondary structures: an effective descriptor for tRNA. *Comput. Appl. Biosci*., **6**, 325-331.

Riccitelli,N.J. and Luptak,A. (2010) Computational discovery of folded RNA domains in genomes and *in vitro* selected libraries. *Methods*, **52**, 133-140.
